# P229: Bacterial flora in burns patients at a tertiary care referral centre in North India

**DOI:** 10.1186/2047-2994-2-S1-P229

**Published:** 2013-06-20

**Authors:** S Appannanavar, N Taneja, G Singh, M Biswal, PS Chari, B Mohan

**Affiliations:** 1 Medical Microbiology, Pgimer, Chandigarh, India; 2 Plastic Surgery and Burn Unit, Pgimer, Chandigarh, India

## Introduction

Bacterial flora in burn patients undergoes a change over a period of time and is dependent upon many factors. Study of burn flora is not only helpful in locating entry of multidrug resistant bacterial strains into the unit’s usual flora but also in determining current antibiotic susceptibilities.

## Objectives

Since no studies are available from India to date that have studied sequential emergence of different microorganisms in burn wound, present study was carried out to study the evolution of bacterial flora in burn wounds and its correlation with invasive wound infection.

## Methods

Patients with 20-70% of total burn surface were enrolled and followed up for their entire duration of stay . Clinical & treatment details were noted. Surface wound swabs were collected on first, third, seventh, tenth and fourteenth day post admission. CDC case definitions were used to define invasive wound infections. Environmental sampling was done every three months.

## Results

Of 215 wound swabs collected from 71 patients, 72 were sterile and 143 yielded 214 isolates. Colonization rates were 33% on first day, 94% on 7^th^ day and 100% by 14^th^ day. 42% swabs grew gram negative bacteria. Overall *Staphylococcus aureus* was the predominant isolate (45%) followed by *Pseudomonas aeruginosa* (13.9%), beta hemolytic Streptococci (9.4%). Maximum invasive infections were seen at the seventh day. A high level of environmental contamination was seen with *S. aureus*, a substantial portion being MRSA.

## Conclusion

Better control of environmental contamination and disinfection along with rigorous hand washing and barrier precautions are recommended to prevent infection of wounds.

## Disclosure of interest

None declared

